# Is radiographic lumbar spondylolisthesis associated with occupational exposures? Findings from a nested case control study within the Wakayama spine study

**DOI:** 10.1186/s12891-019-2994-1

**Published:** 2019-12-26

**Authors:** Yuyu Ishimoto, Cyrus Cooper, Georgia Ntani, Hiroshi Yamada, Hiroshi Hashizume, Keiji Nagata, Shigeyuki Muraki, Sakae Tanaka, Munehito Yoshida, Noriko Yoshimura, Karen Walker-Bone

**Affiliations:** 1 MRC Lifecourse Epidemiology Unit, Southampton General Hospital, Southampton, Hampshire UK; 2 Orthopedic surgery, Wakayama Medical University, Wakayama city, Wakayama prefecture Japan; 3 Orthopedic surgery, Kinan Hospital, Tanabe city, Wakayama prefecture 646-8588 Japan; 4 Department of Preventive Medicine for Locomotive Organ Disorders, 22nd Century Medical & Research Center, Faculty of Medicine, University of Tokyo, Tokyo, Japan; 5 Arthritis Research UK/MRC Centre for Musculoskeletal Work and Health, Southampton General Hospital, Southampton, Hampshire UK; 6 Department of Orthopedic Surgery, Sensory and Motor System Medicine, Graduate School of Medicine, University of Tokyo, Tokyo, Japan

**Keywords:** Spondylolisthesis, Occupation, Agricultural /driving

## Abstract

**Background:**

To explore the relationship between radiographic spondylolisthesis and occupational factors in a case-control study nested within the Wakayama Spine Study (WSS).

**Methods:**

The WSS is a cross-sectional observational study amongst Japanese adults. All participants completed a lifetime occupational history and underwent X-rays of the lumbar spine (L1-S1) according to a pre-defined protocol. One trained surgeon graded the presence of a spondylolisthesis based upon ≥5% anterior or posterior slip at one or more levels. Cases, with lumbar spondylolisthesis, were compared with controls without, for their principal occupation and occupational exposures.

**Results:**

In total, data were available for 722 adults (245 men and 477 women), mean age 70.1 (range 53–93) years. According to the pre-defined radiographic criteria, 117 were defined with spondylolisthesis (cases), leaving 605 controls. Cases were not significantly different from controls for age, gender, BMI, smoking or alcohol intake. However, cases were more than twice as likely to report occupational driving ≥4 h/day (OR 2.39, 95% CI 1.08–5.27) after adjustment for age, gender and BMI. Additionally, after stratification by age using 75 years as a cut-point, cases were more than 3-fold more likely to report having worked in the agricultural/ fishing industries (OR 3.47, 95% CI 1.29–9.29) among those aged < 75 years. A reduced risk of being a case was associated with climbing slopes/steps and walking.

**Conclusions:**

A history of occupational driving and working in the agricultural/fishing industry were associated with radiographic spondylolisthesis in this cross-sectional population study. This finding requires further evaluation in longitudinal studies.

## Background

Spondylolisthesis describes the anterior or posterior migration, or slip, of one vertebra in relation to the next caudal vertebra. The first-line investigation for spondylolisthesis is lumbar spine radiography, studies of which have shown that the characteristic changes are rare below aged 50 years but that the prevalence increases sharply with age affecting as many as 15% of men and more than 50% of women aged 66–70 years [1–3]. It has been demonstrated that the severity of radiographic changes is associated with the risk of symptoms of pain and disability [1]. Disability arises from any combination of low back pain, neurogenic claudication and radiculopathy, associated with disc degeneration, compromise of the canal and central spinal stenosis and/or foraminal narrowing [4]. An incidence rate as high as 4.1% has been estimated in the general population [5]. For the most severe symptoms, surgical management is indicated [6] and rates of surgery for this condition are increasing [7]. Given steady increases in healthy life expectancy, the burden of this condition is likely to increase.

From a public health perspective, understanding the epidemiology and risk factors for this condition could enable us to understand factors which determine longitudinal progression and facilitate the development of preventive strategies to reduce the impact of this disabling condition. To date, age, female gender, ethnicity, hormonal factors (menopause, pregnancy), height, BMI, pelvic and spinal structural factors have been implicated [1, 8] but most of these factors are not, unfortunately, modifiable. Sports and sporting activities, particularly gymnastics, have been associated in some studies [9–13]. Therefore, It has been suggested that more epidemiological studies to define potentially modifiable environmental factors are indicated [14].

There has been evidence for some time that physical workplace exposures are associated with an increased risk of low back pain [15] and degenerative low back pain in particular [16, 17] and therefore, occupational factors could be implicated in spondylolisthesis but there has been limited research to date. The one published study among helicopter pilots described an increased risk of lytic spondylolisthesis [18] and a study among taxi drivers reported an increased risk [14]. More recently, Mariconda and colleagues reported an increased association with self-reported “heavy workload” but not with heaviness of self-reported loads lifted at work or occupational driving and reported a reduced risk associated with “lifetime occupational exposure” and prolonged occupational standing [19].

Therefore, we carried out a case-control study to investigate the association of occupation and occupational activities with radiographic spondylolisthesis among a population sample of older adults in the Wakayama Spine Study (WSS).

## Methods

### Hypothesis

That occupation and occupational factors are associated with an increased risk of radiographic spondylolisthesis.

### Participants

Under the approval of our institutional review board, the present study, entitled the Wakayama Spine Study (WSS), was performed with a sub-cohort of the Research on Osteoarthritis/Osteoporosis Against Disability (ROAD) study. The ROAD study was initiated as a nationwide, prospective study of bone and joint diseases in population-based cohorts. A detailed profile of the ROAD cohort has been previously reported [20, 21]. Therefore, in brief, the ROAD study included 3040 inhabitants (1061 men and 1979 women) aged 23–95 years recruited from resident registries in three communities. ROAD included an urban community, Itabashi-ku, but the WSS, which for convenience was the sampling frame for the current study, only included participants from the two rural communities near Wakayama: Hidakagawa and Taiji. Hidakagawa-cho, is a mountainous community located in the center of Wakayama, which had a population of 11,300/330 km^2^ with 29% of jobs in primary industries (agriculture, forestry, fishing and mining), 24% in the secondary industries (manufacturing and construction), and 47% of jobs in the service industry. Taiji-cho, is a seacoast community located south of Wakayama, with a population of 3500/6 km^2^. In comparison with the above, 13% of the Taiji population work in the primary industries, 18% in the secondary industries and 69% work in the service industry. The ROAD study team made a second visit to Hidakagawa and Taiji between 2008 and 2010. Of the inhabitants who participated in this second visit, 1063 volunteers were recruited for MRI. Fifty-two of these declined to attend the examination, and the remaining 1011 were registered in the Wakayama Spine Study. All participants provided their written, informed consent for the MRI examination. Participants who had sensitive implanted devices (such as a pacemaker) or other disqualifiers were excluded. In total, 977 participants underwent lumbar spine MRI. Ten participants who had undergone a previous lumbar operation were excluded, and 29 participants who were younger than 40 years were excluded. All participants in the WSS were invited to complete an interviewer-administered questionnaire which included 400 questions about demographic factors, lifestyle factors, occupation, and occupational exposures and underwent lumbar spine radiographs and anthropometric measurements. Everybody was eligible to participate, regardless of age, gender and symptoms at baseline, providing that they could give written, informed consent and were able to complete the questionnaire and undergo spinal radiography (pregnant women were excluded). Complete radiographic and occupational data were available for 722 participants (245 males, 477 females), mean age 70.9 years, range: 53–93 years.

The study was approved by the ethics committees of the University of Tokyo and the Tokyo Metropolitan Institute of Gerontology.

### Occupation and occupational activities

A lifetime occupational history was collected alongside details of 7 types of specific work exposures: sitting on a chair for ≥2 h/day; standing for ≥2 h/day; kneeling for ≥1 h/day; squatting for ≥1 h/day; driving for ≥4 h/day; walking ≥3 km/day; going up and down stairs ≥30 floors/day; climbing up slopes or steps for ≥1 h/day and; lifting loads weighing ≥10 kg at least once a week. For the current study, the information on occupational title and exposures was derived from the respondent’s principal occupation (that in which the participant had worked for the longest duration). For comparison, occupations were grouped according to the nature of work as follows: Clerical/technical; agricultural / fishermen; factory/construction; clinical / housekeepers / shop workers / hairdressers / dressmakers; teachers and “other” (for all remaining types of work).

### Assessment of lumbar spondylolisthesis

Lumbar spine radiographs were performed according to a standardised protocol to include the intervertebral levels from L1-L2 to L5-S1. Anteroposterior and lateral radiographs of the lumbar spine were acquired with patients in a standing position. The radiographs were all read without the knowledge of participant symptoms, occupational exposures or function by one experienced orthopaedic surgeon (YI). In line with other epidemiological studies of radiographic spondylolisthesis [22–24], the %slip was calculated as the distance of sagittal translation between adjacent vertebral endplates. A patient was defined with spondylolisthesis if they had a slip ≥5% anteriorly or posteriorly at any lumbar level on the lateral views [22–24].

### Statistical analysis

Participants’ demographic and lifestyle characteristics were summarized using means (SDs) where normally distributed and medians (inter-quartile ranges, IQRs) when not and counts (%) separately for those with spondylolisthesis (cases) and those without (controls). Differences in categorical and continuous characteristics between cases and controls were assessed using chi-squared and *t*-tests, respectively. The effects of type of occupation (using clerical/technical experts as a baseline category), and occupational activities on spondylolisthesis were assessed using logistic regression modelling, before and after adjusting for demographic characteristics, and were summarized by odds ratios (ORs) and 95% confidence intervals (CIs).

As the main focus of this study was to explore the association between occupational factors and spondylolisthesis, and many of the older participants had stopped working as much as 20–30 years prior to their X-ray, we repeated the analyses separately for those < 75 and ≥ 75 years of age, allowing a decade after retirement. Statistical analyses were performed using Stata V.12.1 (StataCorp, College Station, Texas, USA).

## Results

Firstly, the inter- and intra-observer reliability of the radiographic assessment of spondylolisthesis was assessed in two sub-studies. For intra-observer reliability, the orthopaedic surgeon re-assessed a random sample of 50 lumbar spine radiographs a month later, blinded to his original assessment and in a different order. A kappa of 0.85 was obtained for intra-observer agreement. Secondly, in a different sample of 50 randomly-selected radiographs, another experienced observer (SM) assessed the % slip at levels L3–5 for 150 levels of radiographs and obtained similarly excellent levels of inter-observer agreement (kappa =0.83) for the presence/absence of a slip.

In total, 117 (16.2%) of participants were defined with spondylolisthesis (cases) and the remaining 605 individuals as controls (Table 1). There were no differences between cases and controls in terms of sex, age, BMI, usual walking speed (as a proxy for physical function), smoking and alcohol.

**Table 1:** Characteristics of cases with spondylolisthesis and controls

	Cases (*N* = 117)	Controls (*N* = 605)	*p*-value
Sex			
Males	35 (29.9%)	210 (34.7%)	0.316
Age (mean (SD))	71.5 (9.9)	70.8 (9.9)	0.505
BMI (mean (SD))	23.7 (3.5)	23.1 (3.4)	0.069
Usual walking speed (median (IQR))	5 (5–6)	5 (5–6)	0.759
Smoking			
No	108 (92.3%)	556 (91.9%)	0.823
Alcohol			
No	84 (71.8%)	432 (71.4%)	0.932

Table 2 shows the associations between occupational group and occupational activities amongst cases as compared with controls. In the unadjusted analyses (Model 1) no association was found between spondylolisthesis and working in any occupational group (agricultural/fishermen; factory/construction; clinical / housekeepers / shop workers / hairdressers / dressmakers; teachers or “other”), as compared with the referent clerical/technical workers.

**Table 2:** Comparison of associations with occupation and occupational activities of cases of spondylolisthesis with controls

	Total N	Cases	Controls	Model 1			Model 2			Model 3		
				OR	95% CIs	P	OR	95% CIs	P	OR	95% CIs	P
Occupational group												
Clerical / technical experts	197	29	168	1.0			1.0			1.0		
Agricultural / Fishermen	105	16	89	1.04	(0.54,2.02)	0.9	1.01	(0.50,2.01)	0.99	0.99	(0.50,1.98)	0.98
Factory/construction	48	10	38	1.52	(0.68,3.39)	0.3	1.49	(0.66,3.34)	0.33	1.46	(0.65,3.27)	0.36
Clinical / Housekeepers / Shop workers / Hairdressers / Dressmakers	207	39	168	1.34	(0.79,2.28)	0.27	1.27	(0.74,2.19)	0.39	1.24	(0.72,2.14)	0.45
Teachers	51	6	45	0.77	(0.30,1.97)	0.59	0.77	(0.30,1.99)	0.59	0.78	(0.30,2.00)	0.6
Other/NA	114	17	97	1.02	(0.53,1.94)	0.96	0.99	(0.51,1.89)	0.97	0.97	(0.50,1.86)	0.92
Occupational activities												
Sitting on a chair ≥2 h/day	347	60	287	1.17	(0.78,1.73)	0.45	1.21	(0.81,1.81)	0.35	1.21	(0.81,1.81)	0.36
Standing ≥2 h/day	574	87	487	0.7	(0.44,1.11)	0.13	0.68	(0.43,1.09)	0.11	0.67	(0.42,1.08)	0.1
Kneeling ≥1 h/day	115	21	94	1.19	(0.71,2.00)	0.51	1.16	(0.69,1.97)	0.57	1.13	(0.67,1.91)	0.65
Squatting ≥1 h/day	159	26	133	1.01	(0.63,1.63)	0.95	1	(0.61,1.61)	0.99	0.97	(0.60,1.58)	0.91
Driving ≥4 h/day	35	10	25	2.17	(1.01,4.64)	0.05	2.49	(1.13,5.49)	0.02	2.39	(1.08,5.27)	0.03
Walking ≥3 km/day	238	35	203	0.85	(0.55,1.30)	0.44	0.82	(0.52,1.28)	0.37	0.81	(0.52,1.28)	0.37
Going up/down stairs ≥30 floors/day	169	20	149	0.63	(0.38,1.06)	0.08	0.64	(0.38,1.07)	0.09	0.62	(0.37,1.05)	0.07
Climbing up slopes or steps ≥1 h/day	94	11	83	0.65	(0.34,1.27)	0.21	0.6	(0.30,1.22)	0.16	0.63	(0.31,1.27)	0.2
Lifting loads of ≥10 kg at least once per week	327	45	282	0.72	(0.48,1.07)	0.11	0.73	(0.48,1.10)	0.13	0.72	(0.47,1.09)	0.12

Model 1: Unadjusted OR; Model 2: Adjusted for age and sex; Model 3: Adjusted for age, sex, and BMI

When considering occupational exposures, cases were more likely to report exposure to occupational driving ≥4 h/day than controls (OR 2.17, 95% CI 1.01–4.64). After adjustment for age and sex (Model 2), the association was if anything stronger (OR 2.49, CI 1.13–5.49) and was robust even after full adjustment for age, sex and BMI (OR 2.39, CI 1.08–5.27) (Model 3). We found no other occupational exposures to be associated, with or without adjustment.

**Fig. 1:**
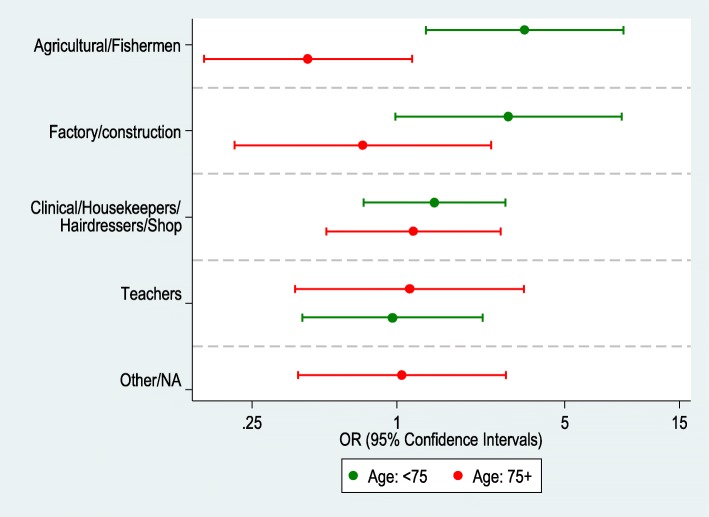
Comparison of the adjusted associations (OR and 95% CIs) among cases with spondylolisthesis, as compared with controls without, among people from the different occupational, stratified by age

Table 3 presents the stratified analysis by age using 75 years as a cut-point. Amongst younger participants (aged < 75 years), an increased association was found amongst cases as compared with controls for working in agricultural/fishing industry as compared with clerical/technical work (OR 3.41, CI 1.32–8.81) (Model 1) and this was robust to adjustment for age, gender and BMI (OR 3.47, CI 1.29–9.29) (Model 2) (Fig. 1). Once again, we found no other association with the other types of occupation (factory/construction; clinical / housekeepers / shop workers / hairdressers / dressmakers; teachers or “other”). No exposures were found significantly associated amongst those aged ≥75 years.

**Table 3:** Comparison of associations of cases of spondylolisthesis with controls by occupational group and occupational activities stratified by age

	< 75 years						> = 75 years					
	Model 1			Model 2			Model 1			Model 2		
	OR	95% CIs	P	OR	95% CIs	P	OR	95% CIs	P	OR	95% CIs	P
Occupational group												
Clerical / technical experts	1.0			1.0			1.0			1.0		
Agricultural / Fishermen	3.41	(1.32,8.81)	0.01	3.47	(1.29,9.29)	0.01	0.43	(0.16,1.16)	0.1	0.43	(0.16,1.17)	0.1
Factory/construction	2.92	(0.99,8.64)	0.05	2.88	(0.94,8.78)	0.06	0.72	(0.21,2.47)	0.6	0.71	(0.21,2.43)	0.58
Clinical / Housekeepers / Shop workers / Hairdressers / Dressmakers	1.44	(0.73,2.84)	0.29	1.33	(0.66,2.69)	0.43	1.18	(0.51,2.71)	0.71	1.07	(0.45,2.59)	0.87
Teachers	No cases											
Other/NA	0.96	(0.40,2.28)	0.93	0.91	(0.38,2.17)	0.83	1.05	(0.39,2.84)	0.92	1.01	(0.37,2.76)	0.99
Occupational activities												
Sitting on a chair ≥2 h/day	0.81	(0.48,1.39)	0.45	0.83	(0.48,1.42)	0.49	1.87	(1.03,3.40)	0.04	1.95	(1.06,3.57)	0.03
Standing ≥2 h/day	0.76	(0.42,1.36)	0.35	0.73	(0.40,1.31)	0.29	0.56	(0.25,1.22)	0.14	0.54	(0.24,1.20)	0.13
Kneeling ≥1 h/day	0.98	(0.46,2.11)	0.96	0.94	(0.43,2.04)	0.87	1.41	(0.69,2.90)	0.35	1.35	(0.65,2.79)	0.42
Squatting ≥1 h/day	1	(0.49,2.03)	1	0.92	(0.44,1.90)	0.82	1	(0.52,1.93)	0.99	0.98	(0.51,1.88)	0.94
Driving ≥4 h/day	2.34	(0.79,6.88)	0.12	2.33	(0.77,7.07)	0.14	2	(0.68,5.86)	0.21	2.6	(0.83,8.15)	0.1
Walking ≥3 km/day	1.32	(0.71,2.47)	0.38	1.25	(0.66,2.35)	0.49	0.53	(0.29,0.98)	0.04	0.55	(0.29,1.02)	0.06
Going up/down stairs ≥30 floors/day	0.38	(0.17,0.88)	0.02	0.36	(0.15,0.82)	0.02	0.97	(0.49,1.91)	0.92	0.99	(0.50,1.97)	0.97
Climbing up slopes or steps ≥1 h/day	0.89	(0.19,4.07)	0.88	0.98	(0.21,4.56)	0.98	0.55	(0.26,1.18)	0.13	0.56	(0.25,1.23)	0.15
Lifting loads of ≥10 kg at least once per week	0.88	(0.51,1.53)	0.65	0.88	(0.50,1.55)	0.65	0.55	(0.30,1.00)	0.05	0.57	(0.31,1.04)	0.07

Model 1: Unadjusted OR; Model 2: Adjusted for age, sex, and BMI

Exploring occupational exposures stratified by age, we found a reduced odds ratio for being a case amongst those reporting exposure to climbing up slopes or steps for ≥1 h/day in those aged < 75 years with and without full adjustment (Model 1: OR 0.38, CI 0.17–0.88; Model 2: OR 0.36, 0.15–0.82). Amongst the older participants (aged ≥75 years) cases were more likely to report sitting on a chair for ≥2 h/day in the unadjusted (Model 1) (OR 1.87, CI 1.03–3.40) and adjusted models (OR 1.95, CI 1.06–3.57) (Model 2). Additionally, reported walking ≥3 km/day at work conveyed a reduced risk (OR 0.53, CI 0.29–0.98) in the unadjusted analyses but this association became non-significant after full adjustment (Model 2).

## Discussion

The results of this study suggest that cases reported a more than doubling of the risk of exposure to occupational driving ≥4 h/day (adjusted OR 2.39, 95% CI 1.08–5.27) in unstratified analyses. Stratification of the analyses by age provided additional insight as, amongst those aged < 75 years, we found that cases were more than 3-fold likely to report exposure to heavy manual work in the agricultural/fishing industries (adjusted OR 3.47, 95%CI 1.29–9.29). Spondylolisthesis cases were more likely to report exposure to driving ≥4 h/day in the stratified analyses but these did not attain statistical significance. However, spondylolisthesis was associated with an almost doubling of risk of reporting sedentary work ≥2 h/day amongst those aged ≥75 years (OR 1.95, 95% CI 1.06–3.57). Additionally, we found that there was a negative association with spondylolisthesis (OR 0.36, 95% CI 0.15–0.82) amongst those aged < 75 years who reported that they climbed up stairs and slopes ≥1 h/day and a similar negative association was seen with walking ≥3 km/day amongst those aged ≥75 years (OR 0.55, 95% CI 0.29–1.02).

The finding that the associations of spondylolisthesis with agricultural/fisherman become attenuated at older ages (≥ 75 years) is not surprising. The retirement age in many Japanese industries is < 65 years so that, in this older cohort, many participants would have stopped work some years prior to their X-rays. It is clear from epidemiological studies that age, gender and hormonal factors play a role in the pathogenesis in spondylolisthesis and it is likely that the impact of occupational exposures overall will relatively diminish as that of other exposures (leisure-time physical activities) increase as years since retirement increases [1, 8].

The results need to be considered alongside some limitations. First, this is a cross-sectional study so that causal attribution cannot be made. Secondly, the participants in this study were sampled from the general population but not at random. We investigated their representativeness by comparing the study population with the general population of Japan for a key risk factor for osteoarthritis, body mass index (BMI). We found that the mean BMI of the participants was not significantly different from that of the general population of Japan (males: 23.71 (3.41) vs. 23.95 (2.64) kg/m2; females: 23.06 (3.42) vs. 23.50 (3.69) kg/m2). However, the study participants reported a lower prevalence of smoking and alcohol use than that seen in the Japanese general population, suggesting participants might live healthier lifestyles. This may limit the generaliseability of these findings. We also cannot rule out a possible selection bias as volunteers needed to be sufficiently healthy to participate and undergo spinal radiographs and this may have limited the possible involvement of elderly institutionalised adults. As spondylolisthesis is a common cause of impaired mobility in older people and immobility may lead to institutionalisation, this may have created a bias, but if so, the effect would have been to reduce the estimated prevalence of spondylolisthesis. The impact of this on the study results would however, only be biased if we believe that those who were employed in any specific occupation were more likely to be institutionalised than those who had worked in others, which seems unlikely. Occupational exposures were obtained by direct inquiry rather than being inferred from job title. Of course the information is dependent upon recall, but the subjects were unaware of their radiographic findings when they were recalling their occupation and occupational exposures so that a systematic bias is unlikely.

It is a strength of this study that all X-rays were assessed by one highly-trained orthopaedic surgeon (YI). In addition, considerable efforts were made to guarantee the reliability of the readings, including inter-observer and intra-observer studies with a sample of 5% of the X-rays, both of which suggested a very good level of reliability (kappa > 0.8 in both studies).

There is disagreement in the literature as to how best to classify radiographic spondylolisthesis. Two principal types are proposed: lytic and degenerative. Lytic is associated with a pars defect of the vertebral arch and usually causes slippage at younger ages. Degenerative spondylolisthesis has been variously described but usually defines a slip occurring with an intact vertebral arch with/without associated advanced arthritis changes in the facet joints at the same level [8]. A systematic review of risk factors for spondylolisthesis found that the selection of cases for observational studies has differed widely [8], some studies restricting selection of cases to those with a slip at L4–5 but others including a slip at any level. Some, but not all, studies included anterior and posterior slips and some studies selected only symptomatic spondylolisthesis or surgical cases. Selection based upon symptoms was criticized in the review given the lack of conclusive evidence that spondylolisthesis causes low back pain. Given the current lack of clarity about case definition, we chose a case definition which is inclusive and consistent with that of other researchers [22–24]. In particular, it was not our intention to separate lytic from degenerative types of spondylolisthesis but rather to use this large population sample to investigate whether any type of occupational exposure was associated with the outcome, in order to generate hypotheses about causation and potentially develop preventive strategies. This is particularly important with occupational research where there is already an established tradition of putting in place preventive strategies to protect workers if an increased risk is established, often in the absence of information about mechanism of causation (e.g. asbestos and lung disease).

Low back pain associated with manual occupations has long been reported [25]. Degenerative changes at the cervical and lumbar spine have been described with working outdoors, heavy lifting and whole-body vibration [25–27]. An increased risk of disc degeneration on MRI scans has been reported amongst those who had undertaken the heaviest occupational lifting [28] and highest lifetime cumulative lifting load [29]. Moreover, increased rates of radiographic lumbar spondylosis were found amongst agricultural/forestry/fishery workers [26], and an increased risk of vertebral endplate sclerosis amongst concrete reinforcement workers [14]. In another recent analysis within the Wakayama Spine Study, we have shown an increased association of lumbar spinal stenosis with factory and construction work [submitted to Am J Ind Med]. The current study adds to a growing body of literature suggesting that the risk of spinal structural degenerative changes is increased in some occupations. The different occupational risk profiles warrant further investigation but might be explained by the selective mechanical effects of these exposures.

Spondylolisthesis has been previously reported in association with occupational driving and flying. Froom et al. reported a four-fold increased risk of lytic spondylolisthesis amongst helicopter pilots as compared with transport pilots and cadets [17]. Among Taiwanese taxi drivers, longer duration of exposure to driving was associated with an increased risk of spondylolisthesis [18]. Mariconda and colleagues found that, amongst 120 back pain patients with spondylolisthesis, occupational driving was the only factor associated with a greater degree of vertebral slip and other occupational activities (awkward posture, prolonged sitting and prolonged standing) were not [19]. It has been postulated that the mechanism by which driving/flying increase the risk of spondylolisthesis is through exposure to whole-body vibration. Indeed, animal studies found that exposure to continuous quantitative vibration diminished the proteoglycan content in the nucleus pulposus disrupting the integrity of the matrix and increasing the instability of the intervertebral disc [30]. The results of our study support the possibility of an association with occupational driving and of course workers in the agricultural/fishing industries may also be exposed to whole-body vibration in tractors or boats [31, 32].

The evidence about other occupational exposures and spondylolisthesis is conflicting. One study reported an increased risk of symptomatic spondylolisthesis amongst those with a heavy workload and undertaking manual handling of materials [19] but another found no association between risk of degenerative spondylolisthesis and the degree of lifting [33]. A study using the same radiographic definition of spondylolisthesis as in the current study found that, although the prevalence of individuals reporting that their ‘longest occupation involved physical labour’ was higher among men with a slip (10.2%) than those without (8.6%), the differences were not statistically significant [24]. It is interesting that we found an association with occupational sitting but only amongst older participants after stratification by age. Two other studies reported no association [19, 33] but differed importantly in their methodology: one [19] only included back pain patients attending outpatient clinics and willing to undergo MRI whilst the other [33] recruited > 4000 people to a population cohort but included a wider age range (22–93), so that the participants were on average 10 years younger than in the current study. More research is required to explore the impact of prolonged occupational sitting but, as sedentary work is becoming increasingly common with mechanization, this could be an important risk factor for spondylolisthesis. Importantly, our findings of a negative association with reported climbing flights of stairs or slopes ≥1 h/day and/or walking ≥3 h/day may suggest that, if replicated in longitudinal studies, being active during working hours may be beneficial in the long-term prevention of this condition.

## Conclusions

We have shown that occupational driving and working in the Agricultural/Fishery industries is associated with spondylolisthesis in this cross-sectional study. Our results also point to a possible increased risk from sedentary work and reduced risk from work that requires walking or climbing up/down slopes or steps. These findings need replication in longitudinal studies to confirm cause/effect but could have implications for countries that define and compensate ‘industrial injuries’.

## Data Availability

The authors will provide anonymized data on request as long as researchers are qualified to request these data. Data requests can be made to Wakayama Medical University Ethics Committee at wa-rinri@wakayama-med.ac.jp

## Abbreviations

BMI: Body mass index
CI: Confidencel interval
MRI: Magnetic resonance imaging
OR: Odds ratio
WSS: Wakayama Spine Study
